# Ethical considerations in implementing AI for mortality prediction in the emergency department: Linking theory and practice

**DOI:** 10.1177/20552076231206588

**Published:** 2023-10-11

**Authors:** Lena Petersson, Kalista Vincent, Petra Svedberg, Jens M Nygren, Ingrid Larsson

**Affiliations:** School of Health and Welfare, 3694 Halmstad University, Halmstad, Sweden

**Keywords:** Artificial intelligence, emergency department, codes of ethics, ethical theory, healthcare professionals, implementation, healthcare, qualitative research

## Abstract

**Background:**

Artificial intelligence (AI) is predicted to be a solution for improving healthcare, increasing efficiency, and saving time and recourses. A lack of ethical principles for the use of AI in practice has been highlighted by several stakeholders due to the recent attention given to it. Research has shown an urgent need for more knowledge regarding the ethical implications of AI applications in healthcare. However, fundamental ethical principles may not be sufficient to describe ethical concerns associated with implementing AI applications.

**Objective:**

The aim of this study is twofold, (1) to use the implementation of AI applications to predict patient mortality in emergency departments as a setting to explore healthcare professionals’ perspectives on ethical issues in relation to ethical principles and (2) to develop a model to guide ethical considerations in AI implementation in healthcare based on ethical theory.

**Methods:**

Semi-structured interviews were conducted with 18 participants. The abductive approach used to analyze the empirical data consisted of four steps alternating between inductive and deductive analyses.

**Results:**

Our findings provide an ethical model demonstrating the need to address six ethical principles (autonomy, beneficence, non-maleficence, justice, explicability, and professional governance) in relation to ethical theories defined as virtue, deontology, and consequentialism when AI applications are to be implemented in clinical practice.

**Conclusions:**

Ethical aspects of AI applications are broader than the prima facie principles of medical ethics and the principle of explicability. Ethical aspects thus need to be viewed from a broader perspective to cover different situations that healthcare professionals, in general, and physicians, in particular, may face when using AI applications in clinical practice.

## Introduction

Artificial intelligence (AI) is expected to have a pioneering role in improving healthcare^1–3^ and increasing the efficient use of medical resources in the coming years.^4–7^ Globally, the healthcare sector faces challenges in meeting demands on providing care based on changes in individual and population care needs and systemic constraints within the healthcare system.^6,8^ The challenges emanate from a shortage of skilled workers,^1,5,6^ a growing demand for care from an aging population,^1,3,5^ increasing healthcare costs, scarce medical resources, and an inequality in care provision.^3,9^ Improvement processes facilitated and augmented by digitalization, particularly based on AI technology, are thus highly sought after to meet these healthcare challenges.^3,10–12^

AI technology has shown promising potential for outcome prediction, pattern recognition, and diagnostic classification to support clinicians in decision-making during diagnosis and treatment.^4,7,13–15^ Different types of innovative AI technologies for clinical-decision support have been developed^4,16^ and tested in clinical trials, for example, in sepsis care,^17–21^ cardiovascular care,^22–24^ and COVID-19 care^25,26^ to predict stages of disease, assess prognosis, assist decision-making, and predict mortality risk.^19–22,25,27,28^ One potentially effective use of predictive analytics in the emergency department is to support decisions on which patient should be admitted to a hospital ward and which patient could be safely discharged or displaced, optimizing the use of healthcare recourses and the quality of healthcare.^8,20,28–30^ Such use of AI technology would transform current practice in the emergency department and would address current constraints with a heavy workload and stress on healthcare professionals.^8,20,21,31,32^ Overcrowding is a constant and inherent challenge to the emergency department organization and is the result of excessive numbers of patients waiting for diagnoses and treatments and prolonged waiting time. In addition to the problems that the waiting time itself creates for the patients, it also risks delaying care for patients in acute need of attendance^32^ to mortality in the emergency department^33,34^ and to predictive analytics for identifying high-risk patients^35^ and patients with mortality risk that could potentially assist in the provision of early care to those patients who are in most urgent need.^8,32^ It could also help to identify patients who require end-of-life treatment and facilitate adequate person-centered care.^36^ Research has shown that it is highly beneficial to allow patients at the emergency department to communicate their wishes for treatment and care at an early stage.^37^ Improving both the efficiency and quality of decisions in the emergency department through the use of AI technology could thus both assist clinicians in making efficient and systematic decisions^21^ and empower the patients’ autonomy.^37^

Although earlier studies advocate the potential for AI technology to streamline and improve healthcare practice with decreased healthcare costs,^4^ it is still important to carefully consider challenges to its implementation in practice and potential risks in relation to patient safety.^1,13^ Studies of technological aspects related to AI in healthcare have been prioritized in research and have focused on precision, accuracy, system errors, data privacy, potential bias, and the transparency of decision-making processes.^1,13,27,38,39^ However, human factors should also be considered when a complex technology such as AI is to be introduced to a sensitive and critical setting such as healthcare.^4^ It raises ethical and moral issues^4^ and several systemic and legal challenges that refer to routines in practice, professional roles and responsibilities, policy, and legislation.^28,38^ Healthcare leaders describe ethical considerations regarding, for example, trust, responsibility,^40^ and autonomy and justice^41^ in relation to the development and implementation of AI in practice. Clinicians describe ethical concerns regarding changes in the clinician–patient relationship and trustworthy healthcare communication, autonomy, and justice^41^ and the risk for misdiagnosis, missed diagnosis, and lost of trust in the relationship between healthcare personnel and patients.^42^ Statements, recommendations, and guidelines have been put forward on the topic of AI and ethics,^38,43^ and the European Commission^39^ has published guidelines on “ethical and trustworthy AI” to address this emerging concern. These guidelines highlight the need for grounding the use of AI technology in ethical and moral principles in society for it to be trusted by the public and societal institutions. Adhering to the values of not inflicting harm, respecting human autonomy, and standing up for fairness and quality are crucial in any healthcare setting.^38,39,44^ These values are addressed in the four prima facie principles of medical ethics: autonomy, beneficence, non-maleficence, and justice.^10,45^ These principles heavily influence the practice of medicine, nursing, and related education of healthcare professionals. Physicians are taught to use frameworks building on the four principles to analyze any given situation^46^ concerning patient care and treatment.^10,45^ A fifth principle on explicability has been introduced in relation to ethics and AI technology and refers to the trustworthiness and the need for proper explanation of issues surrounding AI^39^ and the transparency of the AI algorithm's decision-making processes.^43^

In the case of improving efficiency and quality of decisions in the emergency department through the use of AI technology, it is thus important to understand how the four principles of medical ethics and the additional principle of explicability influence how professionals relate to the implementation of the AI application in clinical practice. Understanding the ethical challenges around implementation is particularly essential when introducing AI technology in a chaotic setting such as an emergency department, where many critical decisions are made.^34^ There is also, however, uncertainty about whether other ethical aspects and principles are relevant to the challenges that may arise when implementing AI in clinical practice,^38,47,48^ and there is a need for research that focuses on ethics of medical AI and research that opernationalize ethical concepts and terminologies.^41^ In addition, the use of AI in emergency departments can change the role of the clinicians,^8^ and it is thus important to explore healthcare professionals perspectives regarding this subject. Against this backdrop, the aim of this study is twofold: (1) to use the implementation of AI applications to predict patient mortality in emergency departments as a setting to explore healthcare professionals’ perspectives on ethical issues in relation to ethical principles and (2) to develop a model to guide ethical considerations in AI implementation in healthcare based on ethical theory.

## Methods

### Study design

This study used an abductive qualitative design meaning that the data analysis is a combination of inductive and deductive approaches where the researcher moves between the two approaches during the analyse process. An abductive approach means that the researchers use an inductive (explorative and empirically)-driven analysis of collected data in combination with a deductive approach (theoretical alignment) where the researcher uses the theory as a framework to guide the interpretation of the data.^49^ It was relevant to use an explorative and empirically driven approach in combination with theoretical alignment to expand the understanding of both reality and theory in order to gain deeper insights and understand new aspects of ethical issues for healthcare professionals when implementing AI in practice. The theoretical alignment was based on ethical principles^39,50^ for exploration of healthcare professionals perspectives, and in addition, the ethical theories of virtue, deontology, and consequentialism^51,52^ were used to develop a model to guide ethical considerations in AI implementation in healthcare. To ensure trustworthiness, the study is reported in accordance with the Consolidated Criteria for Reporting Qualitative Research 32-item checklist.^53^

### Setting and participants

The participants were recruited from two emergency departments in the southern part of Sweden where the county council has developed an AI application with the purpose of predicting the risk for unexpected mortality within 30 days after visiting an emergency department.^36,54^ The participants had not yet worked with the AI application at the time of the study, but it was presented to the participants as a potential technology for implementation and use in everyday practice in their clinical settings. A snowball sampling procedure^55^ was used to recruit interviewees, based on prior knowledge and relevance in relation to the study aim and context. At the end of each interview, the interviewee was asked if they could suggest any colleague that we could interview, and the sampling procedure continued until no further individuals within the setting were identified. The recruitment started at a high organizational level and continued organically until no further informants were suggested representing new roles or perspectives. Seven healthcare managers and eleven healthcare professionals connected to the two emergency departments participated in the study (see Table 1). This number falls within the established range for workplace studies and is sufficient to generally achieve saturation in qualitative inquiries, particularly in studies involving semi-structured interviews.^56^ Some interviewees had additional inscriptions to their professional titles as they were also working with other areas of responsibility in their clinic. They all had experience with patient care and clinical work despite some of them now working in management. The broader definition of “healthcare professional” will be used in the study to include both clinicians (e.g. physicians and nurses) and managers. While transcribing the recorded interviews, the identities of the informants were anonymized, which consequently led to a compromise in terms of contextualization. As a result, we were unable to include supplementary details about the sample profile, such as age and work experience.

**Table 1.** Participants’ characteristics (*n* = 18).

**Role**	Managers	2
	Chief physicians	3
	Nursing managers	2
	Specialist physicians	5
	Emergency physicians	3
	Registered nurses	3
**Sex**	Men	9
	Woman	9

### Data collection

The data collection was conducted between September 2020 and January 2021. Two male researchers (DT, trained in Management Research, PhD, and FG, trained in Innovation Research, PhD) conducted semi-structured interviews using phone and video calls. The researchers had no former relations with these healthcare practices. The interviews began with open-ended questions about the participants’ experiences and expectations of implementing new AI technology into healthcare. Further questions were asked about the needs, challenges, risks, and consequences of using AI applications to assess the 30-day mortality risk of patients at the emergency department and how a prediction model could affect their professional roles. The interview questions guided the participants to address perspectives, concerns, and considerations in relation to the implementation and use of AI applications. Thus, in the interview guide, we asked questions as follows: Can you describe your role and responsibility? How do you view the challenge of being able to assess the risk of mortality at discharge from the emergency department? How do you see that AI-based mortality prediction at discharge from the emergency department could work in practice? Do you see potential unexpected consequences that could come from AI-based mortality prediction at discharge from the emergency department? However, due to the kind of decisions that arise in relation to the output from such an AI application, it is in some situations, primarily the physicians who will be affected by its implementation. Managers, nurses, and physicians thus described how the professional role of physicians would be affected by the implementation during the interviews. Each interview lasted between 50 and 60 min and the total interview time was 17 h and 10 min. All interviews were audio-recorded and transcribed verbatim.

### Data analysis

We used an abductive approach to analyze the empirical data in this study, consisting of four main steps, 1a, 1b, 1c, and 2. See Table 2.

**Table 2.** Analysis process.

**Aim**	**Step**	**Analysis method**	**Result**
To use the implementation of AI applications to predict patient mortality in emergency departments as a setting to explore healthcare professionals’ perspectives on ethical issues in relation to ethical principles	1a	Inductive analysis	310 meaning units were identified
	1b	Deductive analysis based on the five ethical principles: autonomy, beneficence, non-maleficence, justice, and explicability	Mapping of meaning units based on the five original ethical principles and an empirically developed sixth principle
	1c	Inductive analysis	Codes were compared and 10 subcategories emerged within the six ethical principles
To develop a model to guide ethical considerations in AI implementation in healthcare based on ethical theory	2	Deductive analysis based on ethical theories defined as virtue, deontology, and consequentialism	A model for ethical considerations in AI implementation in healthcare

The interviews were analyzed using abductive qualitative content analysis.^57^ The abductive analysis combines both an inductive and deductive approach and entails going back and forth between them to gain an understanding of the data.^49^ Firstly (Step 1a), inductive analysis was used to approach the data. The transcriptions were read thoroughly to understand the contextualization of the data. The audio files were then listened to while reading through the transcribed text. The initial analyses of the interviews were conducted by identifying and extracting meaning units and phrases with information relevant to the first aim. A total of 310 meaning units were identified and condensed to shorter sentences to clarify the meaning while preserving the core message. In the next step (Step 1b), the condensed meaning units were coded and analyzed using a deductive method by exploring predetermined categories that were based on the four principles of medical ethics (autonomy, beneficence, non-maleficence, and justice)^50^ and the additional principle of explicability presented in the guidelines for trustworthily AI^39^ (see Table 3). The principles of beneficence and non-maleficence were combined into one category due to their opposite relationship and codependence.^50^ Codes that could not be matched with existing principles were placed in a new category formed into a new principle named “professional governance,” describing ethical aspects of implementing AI applications in relation to healthcare professionals’ roles and duties. By using subsequent analysis and an inductive approach (Step 1c), the similarities and differences of the codes were finally compared to identify subcategories to correspond the first aim of the study.^49^ Ten subcategories emerged from the data analysis. Two of these are mainly referred to physicians’ work and the others to the work of all healthcare professionals. This part of the analysis process was conducted by the second author (KV), and three authors (LP, IL, and PS) acted as co-assessors. The analysis was discussed continuously with all authors until reaching a consensus.

**Table 3.** Description of the five ethical principles used for analyzing the data in relation to the first aim of the study.

**Principles**	**Descriptions**
*Autonomy*	The principle of *autonomy* mainly means for the healthcare professional to adhere to the norms that oblige them to respect patients’ freedom of choice and decisions (i.e. respecting their self-determination).^58^ The principle includes beliefs of informed consent, privacy, and responsibility.^46^
*Non-maleficence*	The principle of *non-maleficence* refers to not intentionally creating harm or injury to the patient.^50^ It is commonly associated with the principle of beneficence.^47^
*Beneficence*	The principle of *beneficence* refers to the obligation to make efforts to secure the patient's well-being by acting for their benefit to protect the patient from harm and defend their rights.^47,50^
*Justice*	The principle of *justice* emphasizes fairness and equality among individuals and includes several categories of justice, whereas distributive justice is mainly addressed in medical ethics.^44,50^ Distributive justice is the healthcare professionals’ and society's obligation to distribute healthcare resources fairly, equitably, and appropriately.^47^
*Explicability*	The principle of *explicability* describes the lack of insight into AI-based technology as an additional principle for trustworthy AI^39^ and refers to the need for transparency of the AI algorithm's functionality and data.^43^

The next step (Step 2) in the data analysis reflected a gradual deductive process of matching empirical findings to ethical theories of virtue, deontology, and consequentialism (see Table 4).^51,52^ The purpose was to understand how the healthcare professionals’ ethical considerations in relation to the five ethical principles presented above were related to moral character and basic values (virtue), emphasized duties or rules (deontology), or referred to the consequences of actions (consequentialism). This part of the analysis process was conducted initially by two authors (LP, IL), and one author (PS) acted as co-assessors and then discussed continuously with all authors until reaching a consensus.

**Table 4.** Description of the analytical model used for analyzing the data in relation to the second aim of the study.

	**Virtue**	**Deontology**	**Consequentialism**
Theoretical foundation	Aristotle’s moral theory	Kantian ethics	Mill's utilitarianism
Abstract description	An action is right if it is what a virtuous agent would do in the circumstances	An action is right if it is in accordance with moral rules or principles	An action is right if it promotes the best consequences
Concrete description	Basic values, the intention to perform morally correct actions	Obligations and demands from a professional perspective despite consequences	The consequences for patients—results of actions
Focus	Developing good character, focus on the moral	Obligations, duties, and ideal expectations	Consequences irrespective of the intention

The research group represented an interdisciplinary team with experiences in pedagogy, medical and nursing research, and qualitative methods.

## Results

Firstly, the result of this study describes healthcare professionals’ perspectives on ethical aspects in relation to the implementation of AI applications for predicting the risk of mortality among patients in an emergency department. The findings align well with the predefined ethical principles of autonomy, beneficence, non-maleficence, justice, and explicability. However, one aspect that emerged in the analysis was not readily encompassed within these predefined principles. Professional governance was thus suggested as a new additional ethical principle (see Table 5) in relation to implementing AI applications in practice.

**Table 5.** Overview of the categories and subcategories describing ethical aspects of implementing AI applications to predict the risk of mortality among patients in an emergency department.

**Categories**	**Subcategories**
Autonomy	Conflicts between the availability and sharing of AI-based information
	Considerations of using AI applications in relation to patients’ self-determination
Beneficence and non-maleficence	Considerations of AI applications as a support system for physicians in their clinical practice
	Considerations of AI applications acting in the patient’s best interest
Justice	Conflicts between demands and availability of resources in relation to implementing AI applications
	Considerations of using AI applications to systematically provide equitable healthcare
Explicability	Conflicts about AI applications’ value and user-friendliness in clinical work
	Considerations of the trustworthiness of AI applications
Professional governance	Conflicts between AI-based information and physicians’ experience-based knowledge
	AI-based conflicts of interest within and between healthcare organizations

### Autonomy

The principle of autonomy addresses the ethical aspects of providing AI-based information to the patient and the support from AI applications for patients’ self-governance to make health-related decisions.

#### Conflicts between the availability and sharing of AI-based information

The participants had concerns about sharing information provided by AI applications and, particularly, how the patient and their relatives would feel about receiving information on AI-based prediction risk of mortality. They were anxious that there might be a tendency to overestimate the importance of AI-based information as decision support compared to human knowledge and intuition. Some participants compared the idea of an AI-based prediction of mortality risk to a death sentence if used in practice to support decisions on treatment alternatives and, ultimately, the initiation of palliative care. They were also considering how to react and what to do if the patient refused to acknowledge the information provided by the AI application and opposed the decisions made based on such information.

…I think one could look at it as giving the patient a death sentence… ‘the system said you will not survive for very long, we’ll not do anything, we’ll not bother’. [Participant no. 4]

The participants anticipated that AI-based information would have different effects on different patients. It would thus be essential that AI applications and their use were based on understanding and acknowledging differences across individual patients and patient groups and their medical histories. Some patients might feel safer if provided with more information on their health status, while others might be frightened and even hostile to its use. Another concern that was raised was how to document AI-based information on mortality risk. The participants did not find it appropriate to have it written in the electronic health record, as it would then be easily available to the patients through the Open Notes service. They assumed it would not be well received by patients or their relatives.

It's extremely sensitive data…I don’t think it [mortality points] is a variable that should be presented, just like that, in the electronic health records… I don’t think that is appropriate at all. [Participant no. 17]

The participants also deliberated about how and when information from AI applications should be provided to the patient. They questioned the ethics of automatically screening patients who visit the emergency department for mortality risk. They also questioned if it was ethically correct to assess the mortality risk regardless of the reason for a specific patient to visit the emergency department, for example, if only for a minor health problem or if the predicted mortality risk was irrelevant to the cause of the visit.

#### Considerations of using AI applications in relation to patients’ self-determination

The participants were concerned that patients at the end of life rarely make their own decisions on matters valuable to them regarding their health and treatment, such as choosing whether to do another X-ray or not, or to be admitted to the hospital or not. The participants suggested that AI applications could ideally provide the patients with alternatives to decision options to support them in making decisions, thereby increasing their self-determination.

It would be a win for many patients. Getting your last remaining time, the way you want and not having to go to the hospital, that last time and die in the elevator or in the ambulance, or in the X-ray room or alone in an emergency room. [Participant no. 8]

### Beneficence and non-maleficence

The principles of beneficence and non-maleficence refer to the ethical consideration of AI applications for predicting mortality risk as a support system for physicians, acting on behalf of the patient to provide beneficial outcomes.

#### Considerations of AI applications as a support system for physicians in clinical practice

The participants thought that predictions from AI applications could be used to motivate or justify the physicians’ decision-making process, strengthening, encouraging, and enriching the decision process, and speeding up the care process. It could support by providing hard and reliable facts when sensitive information is presented to patients and relatives. Furthermore, it could assist physicians in underpinning decisions when there is unclear information or when experience is low or lacking.

I’d mentally have it a little easier and maybe dare to say the difficult things… I’d also feel a little more certain, because we always feel a little uncertain about ourselves as well. I think it might be even more important for the younger colleagues. [Participant no. 3]

A reoccurring concern was that palliative patients are often sent to emergency departments even when it causes them more harm than good. The AI applications could in such cases guide the healthcare chain to avoid unnecessary admissions and provide an adequate end-of-life plan for patients. AI applications could also be used as a reminder or warning flag for physicians to take further precautions or actions in their clinical work, both in cases when the patient is more ill than they would have initially thought and when variations or changes are documented in the patient's health.

There is something popping up [in the computer screen] and reminds us to maybe help the patient in a different way…. It will be a help for the treating doctors at the emergency department to think a little differently. [Participant no. 1]

#### Considerations of AI applications acting in the patient’s best interest

The participants were generally optimistic about AI, acting in the patient's best interest. They stated that AI applications could bring awareness and attention to the patient by notifying healthcare professionals that these could be the patient's last remaining days in life. It could thus facilitate opportunities to meet patients’ wishes and provide them with the best possible care. The participants described that healthcare professionals at the emergency department are often preoccupied with saving lives, looking at lab test results, and trying to cure or slow down a disease process, and therefore often overlook seeing the person behind the patient, the pain, anxiety, and worry among the patients and their relatives. AI applications could thus support healthcare professionals to halt, reflect, and focus on the patient instead of on treatment that might not be fruitful or even in line with patients’ needs and desires. The participants believed that sometimes healthcare professionals tend to do too much due to a wish to do good, even if there is a risk of causing more suffering than benefit for the patients and their relatives.

If you could then get a flag in the system that says, ‘the probability that you will succeed with something in this case is so small. So, think of the patient instead, do your best to relieve the symptoms, relieve pain or relieve anxiety or whatever’. [Participant no. 4]

The participants also worried about AI applications’ potential harm to patients. They were worried that it could make errors in its predictions and the consequences of that. They were anxious about patients erroneously being sent home instead of getting admitted and not receiving the treatment they need, due to the information provided by the AI application. They believed there could be conflicting feelings among patients, about whether the AI application would act in their best interest.

What I think is a bit worrying is that if you… do this screening or assessment in the emergency room, and then you come to the conclusion, ‘but you will die at any time’. And then you just get sent home instead. Otherwise, you’d have been taken care of. And I think that the patient can react both positively and negatively about ‘yes, I can get home’. [Participant no. 14]

### Justice

The principle of justice addresses conflicts between the high demands on healthcare professionals and the lack of resources to facilitate the use of AI applications and considerations of providing equitable healthcare when using AI technology.

#### Conflicts between demands and availability of resources in relation to implementing AI applications

The participants perceived that resources are lacking to manage information from AI applications and that there is a risk of conflict between new demands on providing healthcare resources and their limited availability. They feared that an additional workload on healthcare professionals could contribute to an increase in the half-hearted handling of patients and ultimately in an increased risk of unjust treatment.

Predicting someone's death in the emergency department with AI, I see that as being a bit problematic. Because of how emergency departments look like today, we don’t really have the resources to deal with that information. [Participant no. 8]

The participants spoke of the implementation of AI applications needing extra resources and time to be set aside to educate and train the professionals, which they believed would be difficult to accomplish without having to reduce the time that can be spent caring for patients.

… if we want healthcare staff in this type of implementation, it takes time away from the patient…. but we may also need to create new resources to be able to do it. [Participant no. 1]

#### Considerations of using AI applications to systematically provide equitable healthcare

The participants suggested that AI applications could affect the quality of care. They mentioned an existing unfairness today regarding who is and who is not entitled to palliative care and end-of-life discussions. AI technology could potentially identify patients with the most needs and efficiently allocate time for professionals to spend with patients.

You can also get resources much quicker in order to facilitate the time that's left. You may know that the patient can’t go home, but if you know that the patient will not live more than 30 days, then you can get short-term accommodation… you can’t sit and wait… [Participant no. 9]

On the other hand, the participants also feared that AI applications could potentially have a negative influence on the availability of care options due to the predicted risk of mortality and worried that this could lead to unfair treatment of patients with high mortality risk.

You know that you’ll need to fight to get that patient admitted to another hospital because you have no available beds for them […] You maybe get a phone call from an angry relative who says ‘are you crazy, my father is not going to die’. It's going to be very difficult. There’ll be resistance. [Participant no. 4]

### Explicability

Explicability refers to the technological robustness of AI applications and addresses professionals’ considerations and understanding of AI applications and trustworthiness around AI technology.

#### Conflicts about AI applications’ value and user-friendliness in clinical work

The participants questioned the potential value AI applications would add to their clinical work and referred that the use of AI applications must result in a significant improvement for it to be successfully implemented and used in practice.

It depends on what it leads to. Is it just a sort of reminder that ‘Hi, this patient is really ill and will probably die within 30 days. Have you made sure that there's a way for the patient to be able to be at home? Or stay at home or receive symptom relief?’. It all depends on what we do with the information. Will it just be a way to ‘well, then I’ll have to admit the patient’, in order to get this work started in some way or is it something that can be implemented straight away? [Participant no. 13]

The participants described the importance of user-friendly and intuitive AI applications in their intended use. There is a considerable risk that healthcare professionals will not use AI applications if the functionality is too complicated and it is too complex to be applied in practice. This could result in a gap between the organization's belief that these patients are identified and managed with the support of AI applications as part of the information systems and that this occurs in practice. Another issue in relation to the potential value of AI applications is that professionals are already occupied with dealing with many false-positive alarms from other information systems. If the use of AI applications would entail the need to deal with additional false-positive alarms to a high degree, it would both add stress to their work environment and negatively impact the level of use in practice.

It must be very easy to use technically as well. … it should be as smoothly and easily incorporated in our existing working flow … otherwise they (the patients) will be forgotten and nothing will be done, you will not have time and not prioritize them. [Participant no. 4]

#### Considerations of the trustworthiness of AI applications

The result showed that the information from AI applications needs to be relevant and trustworthy for the healthcare professional to rely on its ability. The participants mentioned they needed to know about the technology's functionality and the data used for the risk assessment. They found it problematic to trust it fully since there is an issue with the transparency of the algorithm's functionality. They supposed that healthcare professionals who are more skeptical or unfamiliar with technology might have more difficulties trusting the AI application than those who are used to working with information systems in their daily work.

So, they need to have a pretty big pre-understanding to be able to trust the system. So, I think that is the ‘be-all and end-all’. That they feel safe with that ‘oh that's why it buzzes now’. [Participant no. 8]

The participants expressed a need to identify when there might be an erroneous output from AI applications. The participants were concerned that patients needing palliative care might slip through the system unnoticed. They were also concerned about inflicting unnecessary harm to patients when AI applications provide false-positive results. The trustworthiness of AI applications and their safe use in clinical practice was questioned. Some participants stated their worries about AI not being trained properly, misleading them into believing false information or dismissing essential diagnoses. Another concern was that AI could lead them to fall into the pitfalls of blindly trusting the AI application without making their own assessments.

Some of the diseases we are sometimes afraid of when we take a patient to the emergency department are quite unusual. So, there is a risk that you miss it when the system is learning… So, there is a risk that you miss unusual diagnoses by using AI. [Participant no. 4]

There were also concerns about the public view of the healthcare system if AI applications were introduced for mortality prediction. They were concerned that the public would lose faith in the healthcare system and start to view it negatively. They worried the public would think that AI technologies were only introduced to save money or do something sinister. Additionally, they were concerned that the mass media would add to the public distrust of the healthcare system by portraying them negatively.

You don’t want to present it [the score] to the patients and relatives in such a way, that it seems that our only purpose with this is that we should save money in healthcare or get organ donors, or whatever it may be. [Participant no. 3]

### Professional governance

This principle addresses ethical aspects of the healthcare organization and the healthcare professions. It deals with the ethical dilemma that the implementation of AI applications may change physicians’ professional roles and that their experience-based knowledge and clinical skills may be replaced or contested by AI applications. It also deals with ethical aspects in relation to the need for appropriate alliances in and between healthcare organizations to deliver adequate care.

#### Conflicts between AI-based information and physicians’ experience-based knowledge

The participants were worried that AI applications could, on the one hand, provide information that could be comparable to or better than their experience-based knowledge and, on the other hand, provide information that stands in conflict with their own knowledge. The participants spoke of physicians usually having a hunch about when to anticipate a shorter life expectancy for a specific patient or the progression of an illness. They, therefore, believed that AI applications would only be useful as decision support to existing decision-making processes rather than as an autonomous prediction system.

Will this really make any difference to me and my colleagues or even the patient or is it just someone doing some project work somewhere and needing the data. [Participant no. 11]

The participants wondered if AI applications could fail to evaluate a patient's condition by overlooking some factors physicians consider necessary for an assessment. Furthermore, the participants mentioned that some physicians would find adjusting to using AI applications more challenging due to already-established routines or their general interest or confidence in using technology. However, even if AI applications were utilized in healthcare, they believed there still needs to be physicians who can smell, feel, hear, and see patients’ conditions and use professional knowledge to assess if the information from AI applications is accurate.

So, the system says to ‘do nothing, this patient is much sicker than it looks like, yes she may look healthy but she will not make it’. And then maybe you refrain from carrying out measures that perhaps could haves extended the life of the patient. [Participant no. 13]

The participants were worried about how AI applications would influence their way of making decisions and of losing clinical skills, such as critical thinking and clinical evaluation and they felt that it could be problematic if ethical considerations in healthcare were primarily based on information from AI applications and to a lesser extent on human experience-based knowledge. They also feared that AI would replace their professional knowledge and capability and that there would be a time when physicians would not need to learn specific clinical skills in their education as AI would take these over. In addition, there was a fear of AI being misused by inexperienced physicians and the participants therefore questioned who should have the mandate to use the AI application and highlighted the need for proper training before its use and a systematic implementation plan. Otherwise, there could be a risk that inexperienced physicians make decisions based on information from AI applications that are in contrast to existing ethical principles. They also worried about relying solely on AI for clinical assessments as such knowledge is needed if the AI application or IT systems break down.

The participants believed that a combination of professional pride in regard to ethical aspects in relation to their work and fear of digitalization and automation would hinder them from using AI applications in clinical practice. This was both about losing control and feeling that their professional role would be threatened. However, they did not believe that AI would replace their role completely or to the extent that their jobs would be taken away from them.

Many of my colleagues are afraid of excessive digitalization and excessive automation. Because they think it's a threat to our competence and our professional role etc. [Participant no. 4]

Some participants requested an ethical forum to discuss and evaluate the decisions made by AI applications. They believed its use could result in consequences that they cannot pinpoint at the moment since they lack relevant experience. They highlighted the ethical conflicts that may arise regarding how to talk about dying with a patient due to an AI-based mortality prediction and found it hard to place themselves in such a situation.

#### Conflicts of interest within and between healthcare organizations

The participants expressed that healthcare lacks suitable alliances within and across different parts of the organization, particularly between regional and municipal care and in relation to palliative patients. They mentioned lack of communication, followed by insufficient care plans, as the most significant problem within the healthcare system. This may be an ethical conflict if the information from AI applications shows that a patient should receive palliative care and if it is impossible to provide that due to the lack of resources within the care system. The participants also talked about problems related to legal regulations that could affect their decision-making process and make it more difficult to provide collaborative care across different departments and organizations since decisions are not linked or coordinated. They believed that AI could prevent and manage these challenges.

The participants mentioned that transferring responsibility between different healthcare organizations would be an ethical challenge for healthcare professionals in relation to the use of AI applications for mortality prediction in the emergency department. There would likely be an increased transfer of responsibility toward home care in the municipalities. The participants questioned if there would be sufficient resources to manage an increased in-flow of patients.

It also has a lot to do with the organization. It does not have much to do with the identification of [certain patients], but it has a lot to do with the structures that exist in the community and such. [Participant no. 3]

The participants thought that AI applications could lead to a clash between the need to deal with new information and a lack of routines and care plans to do so with respect to the existing ethical principles. Adequate routines and systematic strategies were considered important to feel safe working with the AI applications.

### An ethical model to support AI implementation in practice

**Figure 1.**
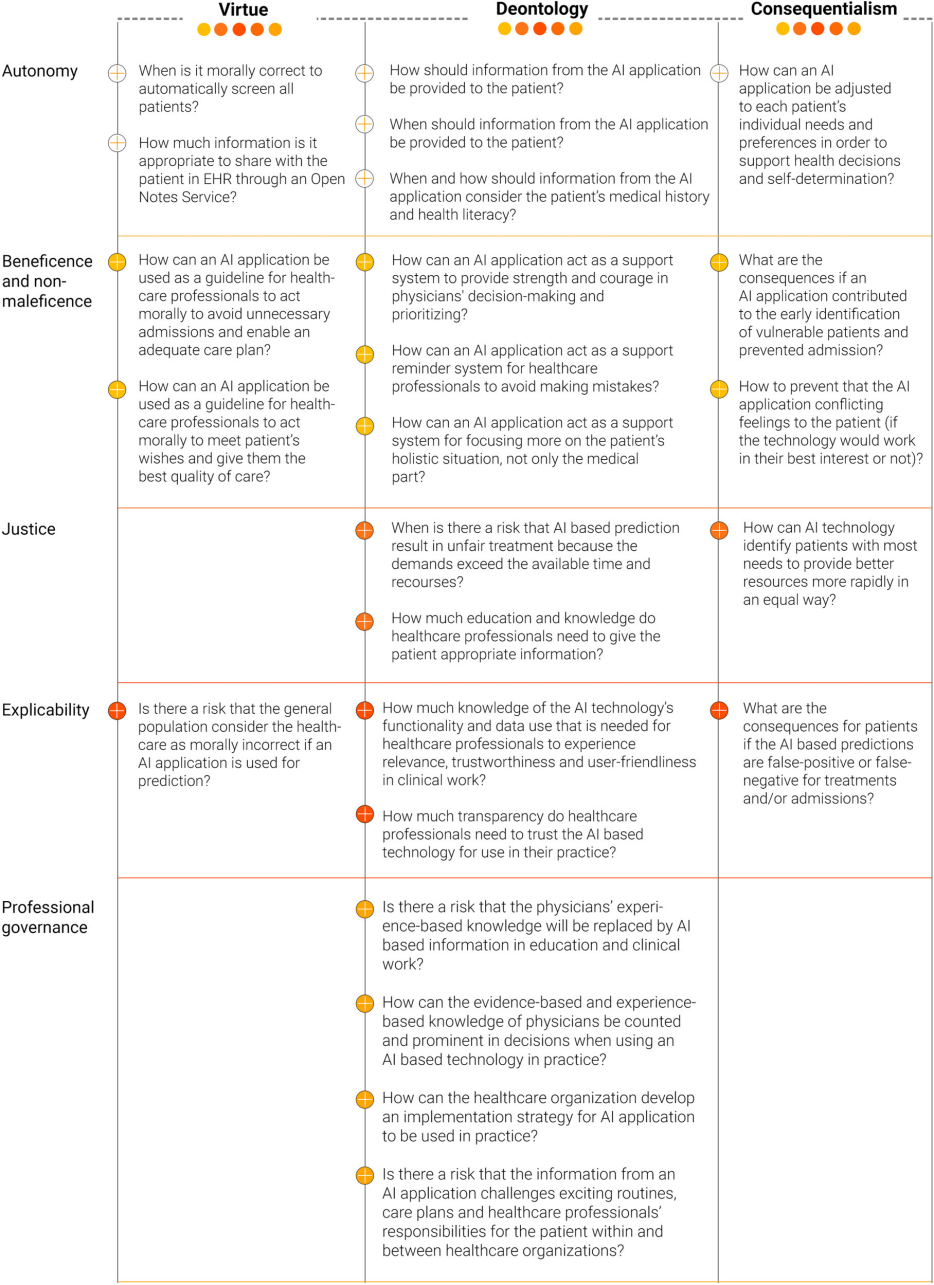
Ethical considerations when AI applications should be implemented in practice.

Based on the findings in Step 1 of the analysis, we propose an ethical model to support AI implementation in practice. This model integrates ethical considerations needed to reflect on and address when the intention is to support AI implementation in practice. An overview of ethical questions abstracted from the participants’ perspectives in relation to ethical principles and ethical theories is given in Figure 1. We can see in the findings that healthcare professionals have considerations related to moral values (virtue) and have many considerations about the duties and rules that can become important to guide the behavior/actions of healthcare professionals in general and physicians in particular (deontology) as well as how these acts are connected to the overall consequences of behavior/actions for patients (consequentialism).

## Discussion

### Principle findings

The findings in this study explore healthcare professionals’ perspectives on the ethical aspects of using AI applications to predict mortality among patients in an emergency department. Even though the healthcare professionals’ perspectives align well with the five predefined ethical principles of autonomy, beneficence, and non-maleficence, justice,^50^ and explicability^39^ this study shows that there is a need to address additional ethical aspects in relation to the implementation of AI systems in practice. A new additional principle, “professional governance,” is thus proposed within healthcare organizations and between different healthcare sectors. This principle addresses ethical conflicts between the healthcare physicians’ experience-based knowledge and information that AI applications provide and the consideration of implementing AI in the organization structure and other healthcare sectors. These aspects do not fit into the four ethical principles described by Beauchamp and Childress,^50^ which focus on patients’ care and treatment rather than the organizational and professional conflicts that emerged as important in our study. Furthermore, these aspects did not fit the principle of explicability addressed in the guidelines for trustworthy AI.^39^ Our findings provide an ethical model demonstrating the need to address the six ethical principles (autonomy, beneficence, non-maleficence, justice, explicability, and professional governance) in relation to virtue, deontology, and consequentialism when AI applications are to be implemented in practice. In the model, when virtue ethics underpin the implementation of AI, healthcare professionals have considerations about the moral aspects and basic values of the use of AI applications. When deontology underpins the implementation of AI, healthcare professionals have considerations about the actions they perform based on the information they have acquired from AI applications and how it adheres to specific duties, roles, and responsibilities for healthcare professionals in general and physicians in particular. When consequentialism underpins the implementation of AI, healthcare professionals have considerations about how to provide better resources more rapidly in an equal way and how AI applications can be adjusted to each patient's individual needs and preferences in order to support decisions, self-determination, and actions in patients’ best interest. The developed model in our study attempts to develop an understanding of ethical considerations needed to reflect on and address when the intention is to support AI implementation in practice. However, this area needs further research to develop even more universal guidelines on ethical issues concerning the implementation of AI in healthcare.^38^

#### The principle of autonomy

The abstracted findings showed that the ethical considerations healthcare professionals experience in relation to autonomy center mostly around the duties and roles that could guide their actions (deontology). The results showed conflicting opinions connected to sharing AI-based information with patients. This finding is inconsistent with respecting patients’ autonomy and acknowledging that healthcare professionals are obligated to disclose necessary medical information and treatment options to enable patients to exercise self-determination.^47^ Patients have the right to make health decisions based on their values and preferences.^32^ They, therefore, have the right to decide whether they want to receive the information from AI applications or not. It is up to the healthcare professional to receive informed consent before disclosing any medical information to the patient.^38^ Nevertheless, there could be an ethical consideration when the patient does not want to know, leaving the healthcare professional in conflict with truth-telling. The principle of autonomy should thus be discussed in the context of trust concerning the relationship between the patient and the healthcare professionals.^38^ The new relationship dynamic could challenge patients’ autonomy since the trust between patients and healthcare professionals is confronted with an additional trust issue in AI applications. The new challenge lies in the transparency of AI technology and the users’ capacity to comprehend the information from AI applications.^42^ On the other hand, using AI applications in clinical decision-making could harm patients’ dignity and autonomy by enforcing a false reality of believing that the AI applications are more knowledgeable. This may undermine shared decision-making between patients and healthcare professionals.^48^ Healthcare professionals thus have a great responsibility to adhere to ethical principles and to guide patients in making informed decisions based on information from AI applications.

#### The principles of beneficence and non-maleficence

Furthermore, this study showed that healthcare professionals questioned the intention of using AI applications for mortality prediction. They consider using AI technology as a support system to act in the patient's best interest and not as a resource to provide the absolute truth. AI applications were believed to be used to justify and motivate physicians’ decision-making to prevent maltreatment. Attempting to use AI technology to support physicians in choosing the best course of action for patients is most centered around virtue, deontology, and consequentialism, which proposes the obligation to provide a net and moral benefit to patients with minimal harm, including acting on behalf of the patient to protect and defend their rights.^46,50^ However, AI raises significant concerns regarding medical responsibility.^14^ Suppose a physician commits liability toward a patient because of a medical error. In that case, they will face the consequences, but using AI technology as part of the decision-making, leaves room for questioning who should be held accountable for a medical error. This is in line with how healthcare leaders reason regarding challenges that arise when implementing AI systems in clinical practice.^40^ There could also be a predicament if the physician did not act upon the suggestion made by AI applications.^48^

#### The principle of justice

The findings address the consideration of equitable opportunities for healthcare, fairness in the disruption of resources and the problem surrounding the shortage of healthcare professionals and time at the emergency departments to implement AI applications for mortality prediction. Varkey^47^ describes the importance of fairness in distributing resources when there is a conflict of interests to provide equal access to healthcare and maximize the benefits from the available resources. These findings could interpret that healthcare professionals are obligated to find solutions for the conflicts between scarce resources and their demands (deontology). However, it is often assumed that using AI algorithms would provide fairer and unbiased outcomes due to not having any personal preferences and human conflicts (virtue), but that is not the case. AI applications have shortcomings similar to human health professionals, as they also weigh some factors over others and could therefore be considered to be biased.^14^ This problem is surrounding the uncertainty of using AI technology.^38,47^ There is a non-intentional injustice when training data-driven AI technology with a skewed and discriminatory dataset. There is a probability of providing false hope or despair, which is contrary to the principles of non-maleficence and beneficence. The conflicts of interest lie in using AI technology trained with biased data that could provide injustice healthcare toward certain patient groups.^59^ The European Commission^39^ addresses that special attention should focus on situations involving vulnerable people concerning the probability of asymmetric power and information.

#### The principle of explicability

One of the critical findings in this study addresses the apprehension toward AI technologies’ accuracy and trustworthiness related to the principle of explicability. The result addressed the participants’ concern about AI applications being misused or misled by the information they provide. In a review study by Keskinbora,^10^ the possibility of misused AI technologies is discussed against the potential of interfering with human rights and peoples’ freedom. Explicability is thus crucial for maintaining trust in AI, meaning that the decision-making process needs to be transparent and understandable to a certain extent. However, patients’ fundamental rights and autonomy must be respected in cases of the “black box” algorithm, where the process is imperceptible and not interpretable by the user due to its complex nature.^10,38^ The concerns about the lack of transparency are in line with the ethics of not harming and acting to benefit the patient.^39^ Furthermore, the participants in this study were apprehensive about trusting the decisions made by AI applications and worried about the consequences they would face if they provided wrong information to the patient. These findings relate to both deontology and consequentialism. The participants reflected on the consequences for patients if AI-based predictions are false-positive or false-negative for treatments and/or admissions. There were also concerns about the profusion of transparency if the mortality points were written in the electronic health record and easily accessed online by patients or their relatives via the Swedish Open Notes service. They found the information to be too sensitive to be disclosed so openly. AI applications might thus lead to additional concerns about transparency in electronic health records. Nevertheless, most studies in recent years have been pro-transparency^60–63^ in that patients own their medical information and thus have the right to access it.^60^ Transparency enables patients to feel empowered and engaged in their care, improving patient satisfaction.^60,62^ However, research also shows that governed individual real-time transparency that visualizes information from the electronic health record through the Open Notes service may have positive effects but can also result in negative trade-offs between transparency and efficiency of the actual practice.^64^ The consequences of transparency are most centered around consequentialism.

#### The principle of professional governance

Furthermore, this study showed that healthcare professionals had ethical considerations about professional governance, mostly in relation to conflicts between actions taken based on AI applications and physicians’ experience-based knowledge (deontology). The participants mentioned threats to the physicians’ professional identity and feared they would lose clinical skills when using AI technology. Physicians’ knowledge challenged by AI technology could thus threaten their professional autonomy and leave them with a sense of conflict toward the profession's core value (virtue) and weakness in their sense of professional influence. Some professionals believed that AI systems would not bring physicians any value in their clinical practice. There was also a sense of rivalry in the field of knowledge and skepticism that AI applications would make better judgments compared to the professional knowledge of an experienced physician. Earlier research has shown that healthcare professionals’ perceptions of the usefulness, value, and relevance of using technology affect the implementation in practice^63,65^ and it could therefore be important to accomplish successful implementation that AI applications are in line with healthcare professionals’ characters, values, and motivations (virtue) and that the morality issues of AI applications to be implemented are centered on actions (deontology) that aim to provide better resources and better effects (consequentialism) from the professionals’ perspectives.

This study further explored a new ethical aspect that addresses the lack of suitable alliances among healthcare professionals, organizations, and other parts of the healthcare sector. It addresses the apprehensiveness toward the lack of strategies and routine plans to face the aftermath of using AI applications, thus related to deontology. The participants suggest that there will be an ethical conflict between the need for applicable regulations and strategies and the already existing absence of well-functioning and synchronizing care between the different healthcare sectors. For example, there was apprehensiveness about whether home care in the municipalities would be able to cope with the aftermath of using AI technology for mortality prediction in the emergency department. There is thus a great need for collaboration between specialists in several care settings when AI applications are implemented in practice^4^ to avoid ethical dilemmas.

### Strengths and limitations

The findings of the study have to be seen in the light of some possible strengths and limitations. AI applications for predicting mortality risk among patients in the emergency department have been developed but not yet implemented in practice. The participants’ perspectives were thus based on AI applications’ potential implementation and use. However, the interviews were rich, and the participants’ perspectives on ethical considerations were highly informative for developing knowledge to guide future implementation processes of AI applications. Only two researchers conducted the interviews, and a limitation could be the risk of bias or subjectivity in the data collection process. However, in order to minimize bias and strengthen the quality, the analysis was conducted using co-assessors and continued discussions within the interdisciplinary research group in all steps of the process. The ethical model to support AI implementation in practice that was developed in the paper could be used as a guideline in this work. The abductive content analysis was an appropriate method to explore a deeper understanding of the ethical aspects from the study subjects’ perspective and to develop a crude model to guide ethical considerations in AI implementation in healthcare.^57^ Dependability is enhanced by the detailed explanation of the methodological description, which allows the reader to repeat the study if necessary.^49,66^ A reflective approach was exercised by the research team throughout the analysis process, which enhanced the study's credibility and confirmability.^49^ The empirical material consisted of 18 semi-structured interviews and a snowball sampling procedure was used and reduce transferability since the probability of them referring to like-minded people is high. This method could affect the diversity of the study data.^66^ However, this approach is preferable since the intention was to study a specific group and deepen the understanding of the phenomenon^67^ and all of the participants worked within the two emergency departments, and there was a variation in the participants’ characteristics. The findings from our study are thus possibly transferable to other emergency departments in Sweden and other countries with similar healthcare systems with the intention to use AI technology to predict mortality.

## Conclusions

This study provides insights from healthcare professionals’ perspectives on the ethical aspects of implementing AI-based technologies to predict mortality in emergency departments. Our findings provide an ethical model demonstrating the need to address the six ethical principles (autonomy, beneficence, non-maleficence, justice, explicability, and professional governance) concerning virtue, deontology, and consequentialism when AI applications are to be implemented in practice. Virtue ethics focuses on healthcare professionals’ considerations about the moral aspects and basic values of the use of AI applications. Deontology focuses on healthcare professionals’ considerations about their actions based on the information they have acquired from AI applications and how it adheres to specific duties, roles, and responsibilities. Consequentialism focuses on healthcare professionals’ considerations about providing better resources more rapidly in an equal way and how AI applications can be adjusted to each patient's individual needs and preferences to support decisions, self-determination, and actions that are in the patient's best interest. The present study has implications for policymakers and healthcare institutions. Based on the findings, a recommendation is to integrate ethical considerations into AI implementation strategies during the development and implementation of AI-based technologies in healthcare. The developed model in our study attempts to increase our understanding of the ethical considerations that need to be reflected on when the intention is to support AI implementation in practice. The model can be used by healthcare managers and project leaders responsible for AI implementation processes, and the questions in the model can serve as a guideline on the ethical considerations that need to be addressed. However, this area needs further research to further develop universal guidelines on ethical issues concerning the implementation of AI in healthcare.
